# Exploring Ancestry‐dependent Regulation of Alzheimer’s disease‐associated genetic risk factors in iOligodendroglia

**DOI:** 10.1002/alz.092403

**Published:** 2025-01-03

**Authors:** Aura M Ramirez, Jihanne J Shepherd, Lauren Coombs, Shaina A. Simon, Sofia Moura, Farid Rajabli, Brooke A. DeRosa, Patrice L. Whitehead, Larry D. Adams, Takiyah D. Starks, Maryenela Z. Illanes‐Manrique, Sergio J. Tejada, Goldie S. Byrd, Mario Cornejo‐Olivas, Briseida E. Feliciano‐Astacio, Liyong Wang, Fulai Jin, Derek M. Dykxhoorn, Anthony J. Griswold, Juan I Young, Jeffery M. Vance

**Affiliations:** ^1^ John P. Hussman Institute for Human Genomics, Miller School of Medicine, Miami, FL USA; ^2^ John P. Hussman Institute for Human Genomics, University of Miami Miller School of Medicine, Miami, FL USA; ^3^ John P. Hussman Institute for Human Genomics, University of Miami Miller School of Medicine, Miami, FL, USA, Miami, FL USA; ^4^ John P. Hussman Institute for Human Genomics, Miami, FL USA; ^5^ Maya Angelou Center for Health Equity, Wake Forest University School of Medicine, Winston‐Salem, NC USA; ^6^ Neurogenetics Research Unit, San Marcos Foundation, Lima Peru; ^7^ Neurogenetics Working Group, Universidad Científica del Sur, Lima Peru; ^8^ Universidad Central del Caribe, Bayamón, PR USA; ^9^ Department of Genetics and Genome Sciences, Case Western Reserve University, Cleveland, OH USA

## Abstract

**Background:**

Genome‐wide association studies (GWAS) in Alzheimer’s disease (AD) are consistently discovering genetic variants linked to the risk of developing this neurodegenerative condition. However, the effect size of the shared associated loci varies across populations as well as each population can have unique associations. This phenomenon could be explained by ancestry‐dependent changes in the genomic regulatory architecture (GRA) influencing the expression of these genes, similar to the effect of different local ancestry on the risk of AD in APOE4 carriers. Thus, understanding of GRA in the context of AD is imperative but currently most GRA data available is predominantly European, limiting our ability to comprehensively interpret the variability associated with AD risk genes across populations. For this study we focused on oligodendroglia, a cell lineage that has been historically overlooked but that is emerging as key players in AD due to their involvement in various pathological processes, including neuroinflammation, oxidative stress, and synaptic dysfunction. Here, we report ancestry‐dependent differences in the GRA of iPSC derived oligodendroglia with African, Amerindian, or European global ancestry.

**Method:**

We obtained PBMCs from individuals with Alzheimer’s disease (AD) or without cognitive impairment, each with over 85% global ancestry of a specific ancestral background. These cells were then transformed into induced pluripotent stem cells (iPSC) and subsequently differentiated into oligodendroglia‐containing 3D neural cultures. On the 76th day of differentiation, we harvested and lysed the cells to isolate nuclei for Multiomic profiling including Single Cell ATAC and Single Cell RNA‐seq, we analyzed the chromatin accessibility and transcriptomes to identify ancestry‐dependent changes genome‐wide and in AD GWAS hits.

**Result:**

We found several AD GWAS hits differentially expressed between ancestries in OPCs and in the more mature oligodendrocyte population (including *APP* and *CLU*) and some differentially accessible peaks associated to some of these genes (predominantly *PRDM7*). Nevertheless, OPCs showed more ancestry‐specific regulation than the more mature oligodendrocytes.

**Conclusion:**

Our findings offer ancestry‐specific understanding of oligodendroglia chromatin changes and gene regulation in the context of AD. These results present a comprehensive perspective on the genetic regulatory architecture of oligodendroglia and constitute a resource for gene identification studies in the African American and Hispanic populations.

